# Production of Poly(3-hydroxybutyrate-*co*-3-hydroxyvalerate) (PHBV) by *Haloferax mediterranei* Using Candy Industry Waste as Raw Materials

**DOI:** 10.3390/bioengineering11090870

**Published:** 2024-08-27

**Authors:** Lorena Simó-Cabrera, Salvador García-Chumillas, Sergio J. Benitez-Benitez, Verónica Cánovas, Fuensanta Monzó, Carmen Pire, Rosa María Martínez-Espinosa

**Affiliations:** 1 Biochemistry, Molecular Biology, Edaphology and Agricultural Chemistry Department, Faculty of Science, University of Alicante, Carretera San Vicente del Raspeig s/n-03690 San Vicente del Raspeig, E-03690 Alicante, Spain; lorena.simo@ua.es (L.S.-C.); carmen.pire@ua.es (C.P.); 2 Multidisciplinary Institute for Environmental Studies “Ramón Margalef”, University of Alicante, Ap. 99, E-03080 Alicante, Spain; 3 Technological Centre of Footwear and Plastic of the Region of Murcia (CETEC) Avda, Europa 4-5, E-30840 Alhama de Murcia, Spain; sg.chumillas@ctcalzado.org (S.G.-C.); s.benitez@ctcalzado.org (S.J.B.-B.); f.monzo@ctcalzado.org (F.M.); 4 Cetec Biotechnology, Avda, Europa 4-5, E-30840 Alhama de Murcia, Spain; v.canovas@ctcalzado.org

**Keywords:** haloarchaea, polyhydroxyalkanoates (PHA), poly(3-hydroxybutyrate) (PHB), poly(3-hydroxyvalerate) (PHV), poly(3-hydroxybutyrate-*co*-3-hydroxyvalerate) (PHBV), candy waste, bioplastics

## Abstract

The haloarchaeon *Haloferax mediterranei* synthesizes poly(3-hydroxybutyrate-*co*-3-hydroxyvalerate) (PHBV) under unfavorable nutritional conditions without the addition of any precursor to the culture, which is an advantage compared to other microbial counterparts able to synthesize polyhydroxyalkanoates (PHA). PHBV is a biodegradable polymer showing physiochemical properties of biotechnological and biomedical interest and can be used as an alternative to plastics made from chemical synthesis (which are not environmentally friendly). The versatile metabolism of *H. mediterranei* makes the use of waste as a carbon source for cellular growth and PHA synthesis possible. In this work, cellular growth and the production and characterization of PHBV using two different types of confectionery waste were analyzed and compared with cellular growth and PHBV synthesis in a standard culture media with glucose of analytical grade as a carbon source. The PHBV granules produced were analyzed by TEM and the biopolymer was isolated and characterized by GC-MS, FTIR NMR, and DSC. The results reveal that *H. mediterranei* can use these two residues (R1 and R2) for pure PHBV production, achieving 0.256 and 0.983 g PHBV/L, respectively, which are among the highest yields so far described using for the first-time waste from the candy industry. Thus, a circular economy-based process has been designed to optimize the upscaling of PHBV production by using haloarchaea as cell factories and valorizing confectionery waste.

## 1. Introduction

In the last decade, there has been a massive increase in waste generation due to processes of anthropogenic origin in general, particularly industrial activities [1]. Worldwide municipal solid waste generation is expected to increase to 2.59 billion tons annually by 2030 and to 3.40 billion tons by 2050 [2]. As an example, over 30% of food is lost or wasted annually, an estimated 1.32 billion tons end up in landfills where food waste goes through a series of bioconversions, which are usually harmful to the environment [3]. Regarding the global concerns facing pollution, current plastic pollution is one of the biggest global concerns, affecting marine and terrestrial ecosystems, animal populations, and human health [4]. It is estimated that around 140 million tons of plastics are produced annually from different sources [5]. If the current production and waste management situation continues, by 2050, around 9000 million tons of plastic waste will be recycled, 12,000 million tons will be incinerated, and 12,000 million tons will have accumulated in the natural environment [6].

Among other processes, food production, processing, packaging, and plastic production and usage are integrated into many processes that finally contribute to global waste production. Production waste is a current concern for the candy industry due to the strict regulations regarding food safety: contamination risks cannot be assumed and companies see their food waste increased to ensure the quality of the final product [7]. The efficiency of resource utilization and waste materials recovery can be enhanced through a circular economy, thus decreasing the emission of fossil carbon during extraction and manufacturing processes. In this context, biotechnological-based procedures can enhance the bioeconomy by using waste generated from the food industry for various applications [3]. 

The growing concern about the effects of plastic pollution on ecosystems and organisms has drawn attention to sustainable materials as an alternative to fossil-fuel plastics. From this point of view, bioplastics are potential substitutes for conventional plastics due to their inherent biodegradability, biocompatibility, and wide range of applications, as well as a production process more friendly to the environment [8]. Polyhydroxyalkanoates (PHAs) are widely recognized as biopolymers due to their applications in the pharmaceutical, medical, and food sectors [9,10,11]. These biological polymers are mainly produced by microbial fermentation processes and are accumulated within microbial cells as granules that serve as carbon and energy storage. The synthesis of these granules takes place when an excess of carbon source is available under nutrient-limiting conditions such as in phosphorus, oxygen, or nitrogen or in a fluctuating pH of the media [12]. Then, the stored PHA granules could be used by microorganisms as a carbon source when the nutrients are limited [13].

Several microbial members of the Bacteria and Archaea domains have been tested during the last two decades for PHA production. Considering the high efficiency of some species able to produce PHAs, those microorganisms are thus revealed as good candidates to be used as cell factories to produce PHA such as poly(3-hydroxybutyrate) (PHB) and poly(3-hydroxyvalerate) (PHV) [14,15,16,17,18]. For example, among halophilic microorganisms, the haloarchaeon *Haloferax mediterranei* and the *Halomonas* species from the bacterial domain have been extensively studied for efficient PHA production. Up to now, *H. mediterranei* has been reported to synthesize PHBV and poly(3-hydroxybutyrate-*co*-3-hydroxyvalerate-*co*-4-hydroxybutyrate) (PHBV4HB), whilst *Halomonas* species can accumulate PHB, PHBV, and poly(3-hydroxybutyrate-*co*-4-hydroxybutyrate) [18,19]. These two genera have been used as model representatives of each domain to study PHA biosynthesis. In both cases, it has been reported that C/N balance, P limitation, overexpression of the genes coding for PHA synthases, and fine-tuning regulation of the TCA cycle are essential to increase the production of PHA [16,19,20,21,22]. In the case of *H. mediterranei,* cells can synthesize PHBV, a more versatile and economically favorable biopolymer than PHB, without the addition of any precursor, which makes this species the most interesting in terms of biotechnological PHBV production compared to its bacterial counterparts [18]. This species can probably use the largest range of single carbon sources (glycerol, glucose, starch, etc.) as well as industrial waste as part of the culture formulation and presents a high growth rate and metabolic versatility [16,23,24]. An important drawback in industrial PHA production by microorganisms is the cost of raw materials, which involves 40–48% of the final costs [25], hence the need to find alternative carbon sources for the growth media promoting the synthesis of PHBV. Only after achieving this aim would the production of PHBV be scalable, economically competitive, and low in time consumption compared to the production of other plastics. The use of waste in the production of PHAs remains poorly explored; considering the lack of homogeneity in the composition of waste, one of the main current concerns is to optimize circular economy-based processes in which the final PHA produced using waste shows the desirable chemical composition and physicochemical properties for targeted industrial and biotechnological applications [10,14,16].

This work aims to study *H. mediterranei* growth as well as PHBV production using waste from the candy industry to optimize a process that is cost-effective, low in time consumption, and environmentally friendly. To the best of our knowledge, this is the first time that confectionary residues are used for this purpose, thus providing a scalable process based on a circular economy.

## 2. Materials and Methods

### 2.1. Candy Waste Used as a Carbon Source

Two different types of solid waste (Table 1) from the candy industry were kindly supplied by the company Vidal Golosinas SA (Murcia, Spain). Both types of waste (Residue 1: R1 and Residue 2: R2) were obtained during the manufacturing of gummies. To use R1 and R2 as carbon sources for microbial growth and PHBV production, R1 was added to the medium before autoclaving. R2 was diluted to 20% in distilled water using a blender and subsequently sterilized using a Steritop/Receiver Flask, Merck KGaA, Darmstadt, Germany filter with a vacuum pump. Then, the solution was added to the autoclaved medium at room temperature. The waste was sterilized differently because R2 cannot be autoclaved, as it becomes caramelized during the high-temperature process, resulting in it no longer being bioavailable. The major polysaccharide identified in both residues was starch (98% starch-rich). Both residues were added to the medium at 1% (*w*/*v*; 1 g/100 mL) as a final concentration.

### 2.2. Microorganism Used and Growth Conditions

The haloarchaeon *Haloferax mediterranei* R-4 (ATCC 33500) has been used as a model organism in this work. Cells were grown in a mineral minimal medium (MM) with potassium nitrate as the sole source of nitrogen. In this study, anhydrous glucose (99%), was used as a carbon source in control cultures according to previous recipes optimized for the growth of this strain [26], while it was replaced by industrial waste from the candy industry to analyze cell growth and PHBV production. The composition of the MM was as follows: 0.7 g NaBr, 0.2 g NaHCO_3_, 6 g KCl, 41.5 g MgCl_2_·6H_2_O, 59.3 g MgSO_4_·7H_2_O, and 234 g NaCl. All the compounds were dissolved in 25% distilled water (25% *w*/*v* salted water (SW)). After autoclaving, 0.005 g/L FeCl_3_, 1% KNO_3_, and 0.001 M Na_2_HPO_4_·12H_2_O/NaH_2_PO_4_·2H_2_O were added. The pH value of the culture media was set to 7.3 using HCl or NaOH. For each condition tested (control and the two candy waste), 1 L cultures were incubated in 3 L Erlenmeyer because of proper aeration (quintuplicates were prepared for all growth conditions). Cultures were inoculated with 10 mL of preadapted *H. mediterranei* R-4 (ATCC 33500) cells (preadapted cells are cells previously grown under the conditions described) grown until the mid-stationary phase of growth (OD_600_ = 1.2). Cultures were incubated in an orbital shaking incubator at 170 rpm and 37 °C until the stationary phase was reached. The growth of the cells was monitored by measuring the OD at 600 nm. The correlation between optical density at a wavelength of 600 nm (OD_600_) and cell number for *H. mediterranei* (Cells/mL = OD_600_ × 9.69 × 10^8^) was estimated using a cell staining protocol with SYBR green (*N*′,*N*′-dimethyl-*N*-[4-[(E)-(3-methyl-1,3-benzothiazol-2-ylidene)methyl]-1-phenylquinolin-1-ium-2-yl]-*N*-propylpropane-1,3-diamine) [27,28].

### 2.3. Analysis of the PHA Granules within the Cells by Transmission Electron Microscopy (TEM)

In total, 20 mL of the cell culture in the stationary phase (OD_600nm_ = 1.95) were harvested by centrifugation at 13,000× *g* RCF for 30 min for TEM analysis. Cells were washed twice with a saline buffer (10% NaCl, 0.1 M sodium phosphate buffer, pH 7.2) and resuspended in a 2.5% (*v*/*v*) glutaraldehyde solution in a saline buffer for primary fixation overnight at 4 °C. Following the primary fixation, the cells were pelleted and washed three times with saline buffer. The cell pellets were then fixed with 1.0% osmium tetroxide in saline buffer (1% *v*/*v*) for 2 h at 4 °C and subsequently washed three times with a saline buffer. A third fixative solution was added to the cells; 0.5% (*w*/*v*) uranyl acetate solution (in Veronal-acetate buffer) and cells were incubated overnight in the dark at 4 °C. The ensuing steps were performed according to the procedure described by Tian and coworkers [29]. Photomicrographs were taken with a Philips Tecnai 12 Electron Microscope (*Carl Zeiss*, Jena, Germany) capable of imaging negatively stained samples between 80 kV and 120 kV and equipped with a megaview III digital camera.

### 2.4. PHA Extraction Method

Biomass was harvested at the stationary phase by centrifugation at 25,861.9× *g* RCF for 30 min. Then, the cells were washed with 10% NaCl and allowed to shake with 0.1% SDS (*w*/*v*) until solubilized. Subsequently, the pellet was separated and frozen at −80 °C to proceed with lyophilization. The lyophilized pellet was treated with hot chloroform at 62 °C for 6 h, followed by precipitation with 10 volumes of pre-chilled methanol. To collect PHBV, the precipitate was centrifuged at 4000× *g* RCF for 30 min and PHA was dried at 40 °C to remove all residual solvent and obtain a constant weight. The pellet, corresponding to raw PHA, was recovered and weighed. The polymer yield was determined by dividing the weight of PHA obtained after extraction by the cell dry weight (CDW), gPHA/gCDW.

### 2.5. Characterization by Attenuated Total Reflect and Fourier-Transform Infrared Spectroscopy (ATR–FTIR)

The spectrum was recorded using a Spectrometer (Bruker Vertex 70, Bruker, Billerica, MA, USA) with an ATR accessory from 400 to 4000 cm^−1^, with a resolution of 4 cm^−1^ and averaged over 32 scans. The extracted polymer has been compared with a commercial PHBV (PHBV Y1000P from Tianam Biopolymer, Ningbo, China).

### 2.6. Nuclear Magnetic Resonance (NMR)

NMR spectra were recorded using a Bruker 400 MHz. All NMR spectra were recorded at 25 °C unless otherwise stated. Chemical shifts (δ) are reported in parts per million (ppm) and referenced to CDCl_3_ (^1^H: 7.26 ppm). The content in the monomer 3-hydroxyvalerate (3HV) was achieved from the integration of the resonance peaks assigned to the methyl protons from the monomeric units 3HV and 3HB in ^1^H NMR, using the equation

$$3HV\left(\%\right)=\frac{I_{3HV}}{I_{3HV}+I_{3HB}}\cdot 100$$

where I_3HV_ and I_3HB_ refer to the integration of the methyl protons of the two monomers (3HV and 3HB, respectively).

### 2.7. Methanolysis and PHA Quantification by Gas Chromatography (GC)

The methanolysis protocol was carried out as described by Kumar et al. in 2017 [30]. In total, 50 mg of lyophilized cells (frozen and dried) were weighed and transferred to a PYREX tube with a plastic screw cap with PTFE-lined rubber. To extract the polymer, the intracellular PHBV underwent a methanolysis reaction that gave rise to the methyl esters of the β-hydroxycarboxylic acids. Then, 2 mL of chloroform, 0.3 mL of 98% sulfuric acid, and 1.7 mL of methanol were added. In total, 0.010 g of benzoic acid prepared in 1 mL of methanol was also added as the internal standard. The tubes were heated at 100 °C for 140 min and cooled at room temperature. Before vortexing the tubes for 30 s, 1 mL of distilled water was added and then the phases were allowed to separate. The chloroform phase (the bottom phase) was collected and transferred to a vial for chromatography analysis. Samples were frozen at this point at −70/−80 °C until analysis. In addition, commercial PHBV with 9% HV (Sigma-Aldrich, St. Louis, MO, USA) was used as an external standard. A curve was made with a PHBV mass of 0.005 g, 0.010 g, 0.015 g, and 0.020 g. These standards must be subjected to the same methanolysis process as the test samples.

Regarding gas chromatography analysis, a Shimadzu gas chromatograph (model 17A) equipped with a flame ionization detector (FID) set at 280 °C was used. The temperature program was set at a temperature of 50 °C for 2 min, subsequently increased from 50 °C to 110 °C at a rate of 20 °C/min, and finally increased to 250 °C at a rate of 20 °C/min. The injector was kept at 250 °C, and the oven was kept at 120 °C. The used column was a VB-WAX (VICI) column, 30 m long, 0.25 mm in diameter, and 0.25 mm thick of the film. The injected volume was 1 μL, with the helium flow adjusted to 1 mL/min, with a total time of 12 min.

To determine the 3HB and 3HV content using the chromatograms, the area values of the 3-hydroxybutyrate methyl ester (3-HBME), 3-hydroxyvalerate methyl ester (3-HVME), and benzoic acid methyl ester (internal standard) peaks were used as follows:

$$Normalized\;area=\frac{3HBME\vee 3HVME\;area}{Internal\;standard\;area}$$

A straight-line pattern was created from the normalized areas to determine the equation of the function (y = a × x + b). 3HB or 3HV weight in the sample was calculated using the equation

$$3HB\vee 3HVweight\in the\;sample=\frac{Normalized\;area\;of\;the\;sample-b}{a}$$

The 3HB or 3HV percentage per cell dry weight (CDW) was calculated using the equation

$$3HB\vee 3HVcontent\left(\%\;per\;CDW\right)=3HB\vee 3HV\;weight\in the\;sample\frac{100}{Cells\;weight}$$

### 2.8. Analysis of the Biopolymer by Differential Scanning Calorimetry (DSC)

A DSC25 TA calorimeter was used to analyze the glass transition of each sample, melting point, and crystallization temperature. Calibration was carried out using zinc and indium standards. The samples were heated from −30 to 185 °C at a rate of 10 °C min^−1^ under N2 flow (50 mL min^−1^). The samples were then held at 185 °C for 3 min before cooling at 10 °C min^−1^ to −30 °C and the cycle repeated. 

The percentage of crystallinity of the samples was calculated according to the following equation:

$${Χ}_{c}\left(\%\right)=\frac{\Delta H_{m}}{\Delta {H^{o}}_{m}}\cdot 100$$

where ΔxH^o^_m_ is the melting enthalpy of the PHBV 100% crystalline and ΔH_m_ the melting enthalpy registered during the first heating scan. The extracted polymer has been compared with a commercial PHBV (PHBV Y1000P from Tianam Biopolymer).

### 2.9. Thermogravimetric Analysis (TGA)

Thermogravimetric analysis (TGA) was performed in a Netzsch TG209F1 instrument TGA 2950. The samples were heated from room temperature to 800 °C at a heating rate of 20 °C/min in a nitrogen atmosphere (nitrogen gas flow rate of 20 mL/min), followed by an isothermal step (800 °C over 20 min) and a second heating to 900 °C at a heating rate of 20 °C/min in an oxygen atmosphere. The derivative of TGA curves (DTG) was obtained using Netzsch analysis software (version 7.0). The extracted polymer has been compared with a commercial PHBV (PHBV Y1000P from Tianam Biopolymer).

## 3. Results

### 3.1. Monitorization of the Cultures Growth

**Figure 1:**
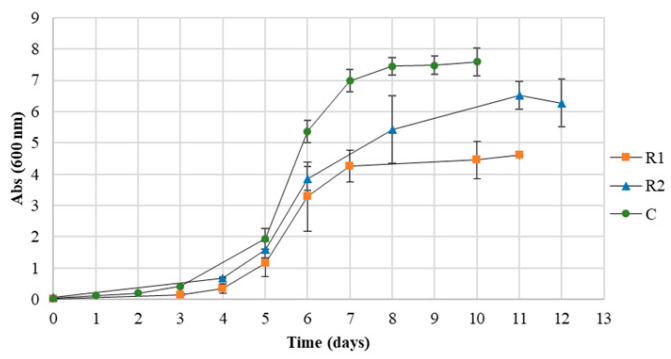
Growth curve (OD_600_) of *H. mediterranei* with the different carbon sources.

Cultures grown with 1% R1 reached the exponential phase around day 4, as did those grown with 1% R2, while cultures with 1% (*w*/*v*; 1 g/100 mL) glucose reached this phase earlier (Figure 1). Faster growth was observed at the beginning of the incubation in the presence of glucose (control media), which is an expected behavior since glucose is a preferred carbon source for *H. mediterranei* cells and its transport processes are simpler to those that could imply preliminary extracellular degradation of the carbon sources, as occurs with starch [31]. Thus, it is possible to think that due to the complexity and heterogeneity of both residues (Table 1), extracellular degradation of the carbon sources could be involved, resulting in a similar behavior in terms of kinetical growth. The R1 cultures were the first to reach the stationary growth phase, specifically on day 7, followed by the control cultures, which took approximately half a day longer, and finally, the cultures containing R2, which reached it on day 8. In addition, the R1 and control cultures showed a more stable and better-defined stationary phase than the R2 cultures. The OD in this phase was 4.6 for the R1 cultures, 6.5 for the R2 cultures, and 7.5 for the control cultures containing glucose. Considering that the chemical composition of the waste could vary among different stocks, up to five replicates were performed to monitor the reproducibility. Chemical analysis of the waste provided by the candy industry and the cellular growth profiles observed indicate that the cellular growth and the PHA production were stable and reproducible.

### 3.2. Analysis of the PHA Granules within the Cells by Transmission Electron Microscopy (TEM)

**Figure 2:**
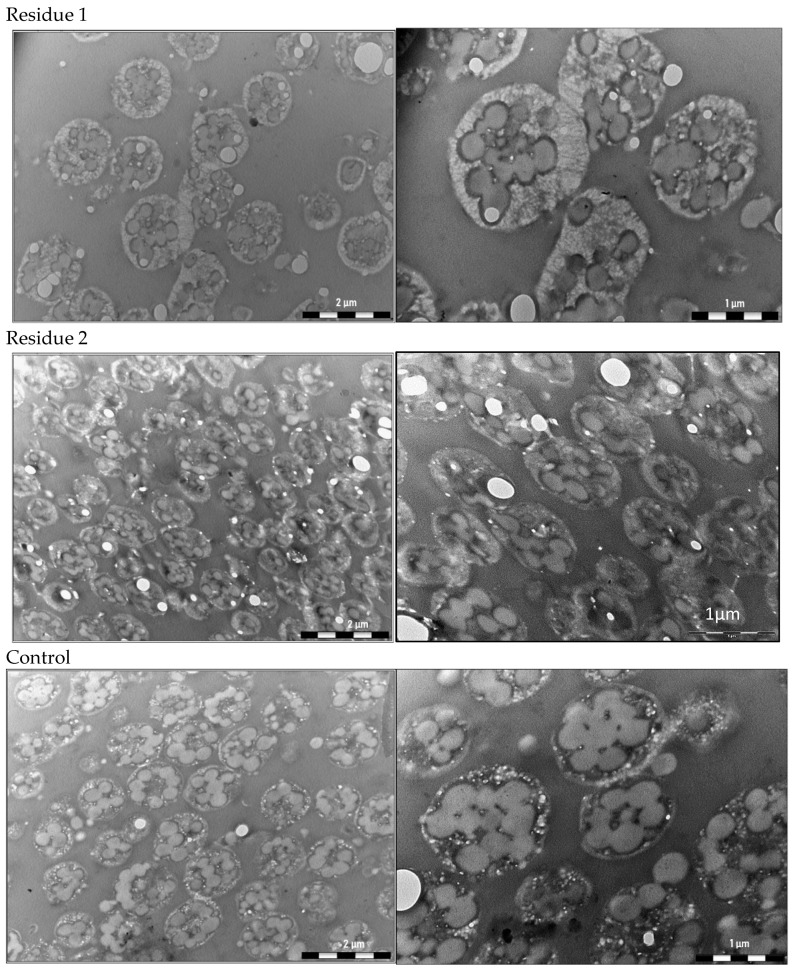
Electron micrographs of *H. mediterranei* cells grown with candy industry residues demonstrate the accumulation of PHA granules. Cells were harvested at the beginning of the stationary phase of growth.

As shown in Figure 2, TEM of *H. mediterranei* cells growing in both residues and harvested at the beginning of the stationary phase of growth revealed the presence of PHA granules. Most of the PHA granules synthesized are spherical and their boundaries are well defined.

### 3.3. PHA Quantification

Total PHA isolation and quantification were conducted as described in the materials and methods section. Cells grown in culture media prepared with each of both residues produced more biopolymer than cells grown in the standard culture; total biomass recovered at the stationary phase of growth was also higher in cultures containing candy residues than in standard cultures. By comparing results obtained from cultures containing the residues (Table 2), it is possible to conclude that cells grown in R2 cultures produced more biopolymer than cells grown in the presence of R1, although the *H. mediterranei* biomass obtained from cultures containing R1 was slightly higher than that the obtained from cultures containing R2. The results related to biopolymer quantification displayed in Table 2 are expressed as grams of PHBV per liter because the analysis summarized in Section 3.4 demonstrated that the biopolymer produced by the cells under the tested conditions was PHBV. R2 is more suitable for PHBV production than R1. *H. mediterranei* can synthesize more polymer in less time, even using glucose as the carbon source. R2 has a lower content of carbohydrates than R1, but a higher content of proteins than the other two substrates, which influences the ratio C/N, thus changing the stress pressure, which affects the cells. In this case, having less stress for growing helps the PHBV accumulation, although this does not happen with the CDW production. 

On the other hand, R1 shows the lowest yields of PHBV production but the highest CDW production. Under this condition, *H. mediterranei* cells have suffered a higher stress due to significant changes in the C/N ratio. Also, R1 does not contain free sugars, which increases cellular stress (the cells must segregate enzymes able to degrade complex polysaccharides like starch to make the sugars more accessible [31]). This process required more time, so *H. mediterranei* took more to produce PHBV.

### 3.4. Characterization of the Biopolymer Produced by H. mediterranei

Considering that PHA constitutes a big group of biopolymers of slightly different structures and compositions, different techniques have been used to identify and characterize the major biopolymer produced by the cells under the tested conditions. Thus, using FTIR, the results revealed that 100% of the biopolymer isolated was a PHBV type. Then, its structure and HV content were confirmed by NMR. Subsequently, HV determination by GC was performed as an additional confirmatory test. Once the chemical composition was elucidated, thermal characterization of PHBV was carried out by differential scanning calorimetry and thermogravimetric analysis. The detailed results obtained from each analysis are described.

#### 3.4.1. Analysis of the Biopolymer by FTIR

**Figure 3:**
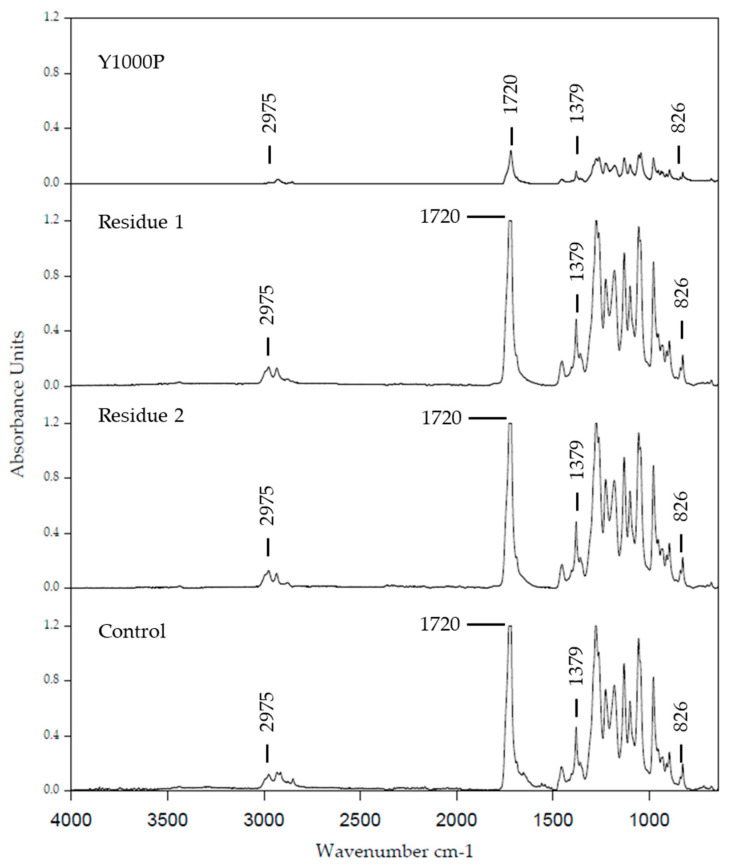
Attenuated total reflectance–Fourier transform infrared (ATR–FTIR) spectra of the PHBV purified from *H. mediterranei* and of the commercial PHBV ENMAT Y1000P (3HV content ≈ 1 mol %).

The purified polymer was analyzed in the solid state by FTIR spectroscopy using ATR sampling methodology and compared with a commercial PHBV (Figure 3). FTIR spectra of the different samples showed an absorption peak centered at 1721 cm^−1^ ascribed to the stretching band of the ester carbonyl bond (C=O). Other absorption bands for the polymer sample obtained under the conditions of this study were found in the range 2924–2856 cm^−1^ (CH, CH_2_ symmetric and asymmetric stretching), 1450–1380 cm^−1^ (C-C stretching), at 1132 cm^−1^ (C-O stretching), and in the range 978–821 cm^−1^ corresponding to C-C deformation. The spectra corresponded to the typical profile of a copolymer PHBV, previously reported in *H. mediterranei* [32]. Unfortunately, the comonomer molar ratio (3HB/3HV) could not be determined by this technique.

#### 3.4.2. NMR and GC

To complete the structural analysis of the PHBV, the polymer composition was investigated by nuclear magnetic resonance (spectra in Supporting Information), and the results are gathered in Table 2. The results corroborate FTIR analysis conclusions, i.e., the biopolymer synthesized corresponded to the PHBV type. The PHBV produced by the cells grown in the control cultures and cultures containing R1 showed higher content of the comonomer 3HV (11.90 ± 0.85% and 12.13 ± 0.85%, respectively) than the biopolymer produced by the cells grown in cultures containing R2 (8.83 ± 0.37%). The concentration of 3HV was also analyzed by gas chromatography. These results differ from GC results because they are complementary and were not performed with the same substrate. In the case of GC, the methodology was applied directly over the biomass, without isolation of the polymer. Conversely, the content in 3HV measured by NMR was inferred from the spectra of the purified materials.

The biopolymer synthesized by cells grown in control cultures accumulated 33.17 mg of 3HB (66.34%, in % per CDW), while the PHBV synthesized by the cells grown in cultures with R1 contained 31.03 mg of 3HB (62.07% in % per CDW). The PHBV obtained from cells grown in the presence of R2 showed the highest amount of 3HB, making up to 48.02 mg of 3HBweight (96.05% of 3HB content in % per CDW).

Regarding the 3HVcomonomer content, the PHBV produced by the cells grown in control cultures showed a higher content of 3HV (5.07 mg of 3HVweight (10.15%)), followed by the PHBV synthesized by cells grown in the presence of R2 cultures (4.68 mg (9.36%)) and finally, the PHBV obtained from cells grown in the presence of Residue (4.32 mg of 3HV (8.63%)) (Table 2) (Figures S1–S3).

#### 3.4.3. Thermal Characterization of PHBV: Differential Scanning Calorimetry and Thermogravimetric Analyses

The thermal properties of the PHBV were investigated by differential scanning calorimetry (DSC) (Figures S4–S7) and thermogravimetric analysis (TGA) (Figures S8–S11). The results are gathered in Table 3. Increasing the amount of the comonomer 3HV gave rise to a decrease in the melting temperature and crystallinity [33]. In this research, the data obtained by DSC and NMR are perfectly correlated, where the copolymer with the highest hydroxybutyrate content, the commercial one, has the highest degree of crystallinity (64.0%). The next polymer with the lowest percentage of hydroxybutyrate is the biopolymer obtained from cells grown with R2, being the next in percentage of crystallinity (52.0%). They are followed in hydroxybutyrate content by the polymer obtained with the control, which has a crystallinity percentage of 48.4% and finally, the PHBV obtained from cells grown with R1 is the one with the lowest hydroxybutyrate content and therefore the one with the lowest crystallinity (47.8%). The crystallinity data do not agree with those obtained by GC, because the percentages of hydroxyvalerate were obtained from the biomass and not from the purified polymers as in the case of NMR. Furthermore, from the melting temperatures of the polymers obtained from R1, R2, and the control, it can be concluded that none of them is close to the melting temperatures of the commercial PHBV with an HV content of 1% and a single melting peak, with a minimum of 176 °C. The polymers isolated from cells grown with both the residues and in control cultures displayed two melting peaks with minimums between 131 and 146 °C, which suggests a random polymeric structure of the monomers [34]. Moreover, the results extracted from TGA thermograms indicate a slight reduction in the thermal stability of the polymer as the content in 3HV increases (T_5%_ and thermal decomposition temperature (T_d_) parameters).

## 4. Discussion

Haloarchaea are extremophilic microorganisms inhabiting salty environments distributed worldwide that have focused the attention of the global scientific community due to their genuine metabolism and their potential applications in biotechnology [35,36]. Among them, species belonging to the genera *Haloarcula* and *Haloferax* (particularly the *Haloferax* genus) are the best haloarchaeal species described from a microbiological and biochemical point of view. As an example of their biotechnological applications, the existence of a peculiar family of repeated DNA sequences spaced by intervening sequences of sizes similar to that of the repeated unit in prokaryotic genomes (SRSR) was first described from the haloarchaeon *H. mediterranei*, which was isolated from Salta Polar saltern ponds [37] Those sequences were later termed CRISPR (Clustered Regularly Interspaced Short Palindromic Repeats) and have constituted a revolution in biomedicine and genetic therapy in terms of genome editing thanks to CRISPR-Cas technology [38]. Regarding other potential applications of *Haloferax* species, the following can be highlighted: the production of several enzymes of interest for industry (dehydrogenases, oxidoreductases, etc.) [39], the production of C_40_ carotenoids highly marketed [24] and C_50_ rare carotenoids with high antioxidant and antitumoral activity [40], the capacity of soil and water bioremediation (heavy metals removal from brines and wastewaters as well as petroleum and petroleum derivatives degradation, etc.) [41], and polyhydroxyalkanoates (PHAs) synthesis (biopolymers of a plastic nature that offer benefits of great interest for various industrial sectors) [16].

The great global problem related to the pollution of the environment by plastics has led to the emergence of a new trend of research, transfer, and development that focuses on the search for new polymers with plastic properties that are biodegradable and whose production follows environmentally friendly processes. In this context, polyhydroxyalkanoates (PHAs) produced by numerous microorganisms are revealed as an alternative to synthetic plastics [10,11,16]. Among the microorganisms able to produce PHAs, extremophiles in general [42] and some haloarchaeal species of the *Haloferax* and *Haloarcula* genera, in particular, produce these biopolymers, accumulating them within the cytoplasm as granules [16,43]. During the last two decades, efforts have been made to (i) characterize the genes coding for the enzymes involved in the synthesis of the PHA as well as the kinetic parameters and structural properties of these enzymes [44,45]; (ii) to study the proteins associated with the PHA granule formation [46,47], and (iii) to explore the use of haloarchaea as cell factories to produce PHA, with these processes being economically profitable and less time-consuming compared to those involving bacterial counterparts [16]. 

The results described in the literature suggest that haloarchaea are good models for producing PHA at a large scale because the content of biopolymers per cell/gr is higher than the average content described from bacterial-based studies. On the other hand, the presence of high salt concentrations in the culture media used for PHA production by haloarchaea makes it possible to avoid autoclaving, thus making the process more economically efficient. Some species of *Haloferax* species produce PHBV as the major PHA, the most marketed bioplastic due to its physicochemical properties, which is another advantage of the use of haloarchaea for this purpose [16,18]. However, the use of haloarchaea to produce PHA on a laboratory scale has revealed two main challenges: (i) although the production of PHA per gram of cell is maximized by optimizing the culture media and/or using mutant strains, the maximum possible production reported in the literature confirm the production of bioplastic using haloarchaea is far from being able to satisfy production needs on an industrial scale and (ii) the optimal production of PHA requires the use of high concentrations of carbon, which makes the cultures and the downstream process more expensive than the conventional production of petroleum-derived plastics [15,16,17,18].

To improve the production of bioplastic and oversee the challenges mentioned, the use of industrial waste as raw materials to grow PHA-producing microorganisms is currently the main aim in combination with the production of new mutant strains and the design of one or two-stage cultivation procedures [48]. Thus, circular economy processes are established, aiming at the valorization of waste and, at the same time, allowing the production of a biomaterial with high added value in the market [16]. 

In the case of haloarchaeal species, different types of waste have been tested to analyze the cellular growth and production of PHA [16]: sludge [49], silkworm excrement [50], olive mill wastewater [48], pretreated vinase [51], hydrolyzed whey [52], petrochemical wastewater [53], date palm sugars [54], rapeseed [55], wasted bread [56], ulva hydrolysate [57], and ricotta hydrolyzed cheese whey [32]. A deep analysis of the literature on this topic reveals that *Haloferax mediterranei* has been the model organism in around 75% of the works reporting original research [16]. This is due to the high efficiency of this species in producing PHBV even in the absence of valerate as a precursor. The use of this precursor is essential to produce PHBV by other microorganisms, an aspect that makes the production of PHBV in these cases unfeasible from an economic and scaling point of view. PHBV shows interesting physicochemical properties for different uses like packaging or biomedical prosthetics and medical instruments, therefore having a microbial species able to directly produce it without the addition of any precursor allows the design of more efficient processes to upscale its synthesis [58,59].

Within this context, this work explores the growth and production of PHA by *H. mediterranei* cells using two types of waste from the candy industry, both mainly starch-rich. The candy market size has been valued at USD 69.20 billion in 2024 and is poised to grow up to USD 83.46 billion by 2029, growing at a CAGR of 3.82% in the forecast period (2023–2030) and being, the largest market from Asia to the Pacific [https://www.mordorintelligence.com/industry-reports/candy-market (accessed on 1 May 2024)]. Due to the legislation applicable in the candy industry and the established quality controls of the products, the magnitude of waste generated in this sector worldwide is high [60]. Therefore, when the design of this research was established, it was concluded that the use of this waste would not only reduce the costs of PHA production by *H. mediterranei* but would also represent a solution for the valorization of waste that is produced on a large scale all over the world. The results obtained indicate that *H. mediterranei* cells can grow in the presence of both candy waste as carbon sources, although the kinetic parameters of the growth were better with R1 than with R2. By comparing the OD of the cultures, including the control culture, it is possible to see that more biomass was produced in the control culture compared to the cultures grown in the presence of R1 and R2 (Figure 1); and R2 was the waste promoting higher production of biomass than R1 (Figure 1). However, the results displayed in Table 2 confirm that the total biomass obtained from R1 cultures was slightly higher than the total biomass produced in R2 cultures and significantly higher than the biomass obtained from control cultures. One explanation for this discrepancy is that the highest values of OD in control cultures may be due to the pigmentation of the cells (they were more colorful than those grown in cultures containing waste and the absorbance of the pigments could interfere with the OD read at 600 nm). The second explanation for this discrepancy could be related to the interference of the PHA granules on OD measurement as described in the literature (since granules generate turbidimetry) as previously mentioned. Consequently, the production of the biomass was also monitored by correlation OD and cell counting as well as by the weight of wet and dry biomass at the time of isolating the biopolymer.

The biopolymer isolated and characterized by GC, NMR FTIR, and DSC from biomass grown using the waste was PHBV-type in both cases. The content of each comonomer (3HB and 3-HV) slightly differs when comparing the composition of PHBV produced by using R1 and R2. In summary, in R1 and control cultures, cells produced PHBV with a higher content of 3HV than cells grown in the presence of R2 (as confirmed by NMR). The higher content of 3HVcomonomer gave rise to a decrease in the melting temperature and crystallinity (Figure 3, Table 2), so the possibility of obtaining PHBV with different proportions of each comonomer opens a new way to design upscaling of PHBV production using candy waste to produce a variety of PHBV showing different degrees of rigidity and consequently potentially uses and different purposes.

To the best of our knowledge, the process optimized here allowed the highest PHBV yield described up to now: 0.236 or 0.983 gPHBV/L with R1 and R2, respectively, which correspond to 23.6% *w*/*w* and 37.8% *w*/*w*. Other studies confirmed a yield of PHB of around 33.4% or a total PHA of around 43% [48]. Many other studies reported similar percentages (between 20 and 56), but in most cases, the percentage corresponds to total PHA or PHB, which makes it difficult to establish an accurate comparison in terms of PHBV; moreover, in some of the reported works, the addition of 3HV precursors was essential to produce PHA [48,49,51,52,54,55,56,57].

Parameters like gPHBV/L and 3HV production are more manageable and accurate to compare between results obtained from different studies. Table 4 shows results from studies in which *H. mediterranei* is the PHBV production microorganism and the carbon source is a waste. The PHBV production observed using R1 and R2 is below the production described in some of these studies, although the 3HV production obtained in this work is in the bibliography range. To this extent, it is worth mentioning that in many of the previously reported works, 3HV precursors were added to improve the production of PHBV, which was not the case in this research.

## 5. Conclusions

This study represents a significant step forward in the quest for sustainable and cost-effective PHBV production by the haloarchaeon *H. mediterranei*. Moreover, waste from the candy industry is valorized. The production of the bioplastic by the cells grown with both residues duplicated (R1) or even triplicated (R2) the total amount of PHBV produced by the cells grown in control cultures, thus opening a new way to design and upscale the production of PHBV coupled to bioremediation processes and circular economy strategies. To the best of our knowledge, this is the first time that candy waste has been used for this purpose, using haloarchaea as cell factories and achieving a significant production of PHBV (up to 0.983 g PHBV/L in the case of cultures containing R2). The physicochemical characteristics of PHBV make this biopolymer attractive for uses such as biomedical utensils (prostheses and surgical utensils). Therefore, although large quantities of PHBV cannot be currently produced by biotechnological approaches, the added value of this bioplastic and the specific uses that it has, such as those mentioned in biomedicine, make the optimization of processes here described interesting and profitable from an economical point of view.

## Figures and Tables

**Table 1:** Nutritional composition of waste (R1 and R2). The nutritional quantification was provided by the company “Vidal Golosinas” S.A. (Spain).

Average Values	Residue 1	Residue 2
Total fat (g/100 g)	0	0
Saturated fat (g/100 g)	0	0
Total carbohydrate (g/100 g)	91	83
Sugars (g/100 g)	90	73
Protein (g/100 g)	0.3	4.4
Salt (g/100 g)	0.01	0.16
Energy/100 g	1558 kJ 374 kcal	1505 kJ 354 kcal
Pictures of each waste	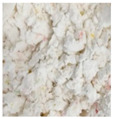	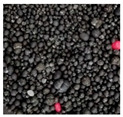

**Table 2:** Cell Dry Weight gram per liter, PHBV grams per liter, PHBV yield, and 3HV% content in *H. mediterranei* cells growing with different residues from the confectionery industry.

Sample	gCDW/L	gPHBV/L	gPHBV/gCDW	3HV% by GC ^a^	3HV% by NMR ^b^	mgPHBV/L
Y100P	-	-	-	-	1 ^c^	-
Residue 1	2.840	0.256 ± 0.166	0.236 ± 0.075	8.630 ± 0.350	12.129 ± 0.849	21.323 ± 3.864
Residue 2	2.600	0.983 ± 0.330	0.378 ± 0.112	9.36 0 ± 0.430	8.832 ± 0.371	66.943 ± 10.912
Control	1.890	0.350 ± 0.095	0.111 ± 0.030	10.150 ± 0.740	11.901 ± 0.853	35.018 ± 3.044

^a^ the analysis was carried out on the biomass; ^b^ the analysis was carried out on the PHBV; ^c^ information provided by the manufacturer.

**Table 3:** Thermal parameters obtained from TGA and DSC measurements.

	TGA		DSC					
Sample	T_5%_ (°C)	T_d_ (°C)	T_g_ (°C)	T_cc_ (°C)	T_mp1_ (°C)	T_mp2_ (°C)	T_m_ (°C)	X_c_ (%)
Y1000P	281.2 (1.7)	298.2 (2.6)	n.d. ^a^	n.d. ^a^	n.d. ^a^	169.9 (0.11)	176.20 (0.68)	64.0 (2.0)
Residue 1	265.5 (2.4)	285.6 (3.1)	−0.41 (0.97)	56.77 (0.99)	132.17 (0.99)	145.89 (0.65)	162.26 (4.77)	47.67 (2.63)
Residue 2	265.8 (0.8)	288.2 (1.2)	−0.10 (1.47)	n.d. ^a^	134.96 (7.41)	145.83 (6.13)	157.55 (0.91)	51.97 (5.56)
Control	261.3 (2.9)	283.3 (4.2)	−0.15 (0.89)	54.46 (10.80)	131.39 (5.35)	141.57 (1.88)	158.66 (1.27)	48.39 (6.49)

Errors are given as standard deviations (values in brackets). ^a^ Not detected. T_5%_: the temperature at which mass loss achieved 5% of the initial mass; T_d_: temperature of decomposition maximum rate; T_g_: glass transition temperature; T_cc_: cold crystallization temperature; T_mp1_: first melting peak temperature; T_mp2_: second melting peak temperature; T_m_: melting peak temperature; X_c_: crystallinity.

**Table 4:** PHBV and 3HV production by *H. mediterranei* using waste as a carbon source.

Waste	gPHBV/L	HV (%)	References
Residue 1	0.256 ± 0.166	12.129 ± 0.849	This study
Residue 2	0.983 ± 0.330	8.832 ± 0.371	This study
Olive mill wastewater	0.2	6.5	[48]
25% pre-treated vinasse	19.7	12.6	[51]
50% pre-treated vinasse	17.4	14.09	[51]
Hydrolyzed whey	12.2 ^1^	6	[52]
Date extract	4.5	18	[54]
75% hydrolyzed rapeseed meal	0.512 ± 0.164	10.000 ± 0.007	[55]
Wasted bread	1.293 ± 0.216	10.78 ± 0.10	[56]
Seaweed hydrolyzed	2.08 ± 0.34	10.6	[57]

^1^ Max. concentration.

## Data Availability

The original contributions presented in the study are included in the article, further inquiries can be directed to the corresponding author.

## Supplementary Materials

The following supporting information can be downloaded at https://www.mdpi.com/article/10.3390/bioengineering11090870/s1: Figures S1–S11. Figure S1. ^1^H-NMR spectrum (400 MHz, 25 °C) of the sample Control; Figure S2. ^1^H-NMR spectrum (400 MHz, 25 °C) of the sample Residue 1; Figure S3. ^1^H-NMR spectrum (400 MHz, 25 °C) of the sample Residue 2; Figure S4. DSC curves of the sample PHBV Y1000P; Figure S5. DSC curves of the sample Control; Figure S6. DSC curves of the sample Residue 1; Figure S7. DSC curves of the sample Residue 2; Figure S8. Thermogram of the sample PHBV Y1000P; Figure S9. Thermogram of the sample Control; Figure S10. Thermogram of the sample Residue 1; Figure S11. Thermogram of the sample Residue 2.
