# Supplementary material for: Production of Poly(3-hydroxybutyrate-co-3-hydroxyvalerate) (PHBV) by Haloferax mediterranei Using Candy Industry Waste as Raw Materials
Source: Bioengineering (Basel). 2024 Aug 27;11(9):870. doi: 10.3390/bioengineering11090870 (PMC11429114; doi:10.3390/bioengineering11090870)
Supplement: Supplementary file 1 [file bioengineering-11-00870-s001.zip › bioengineering-3106952-supplementary.pdf]

Supporting Information

NMR spectra

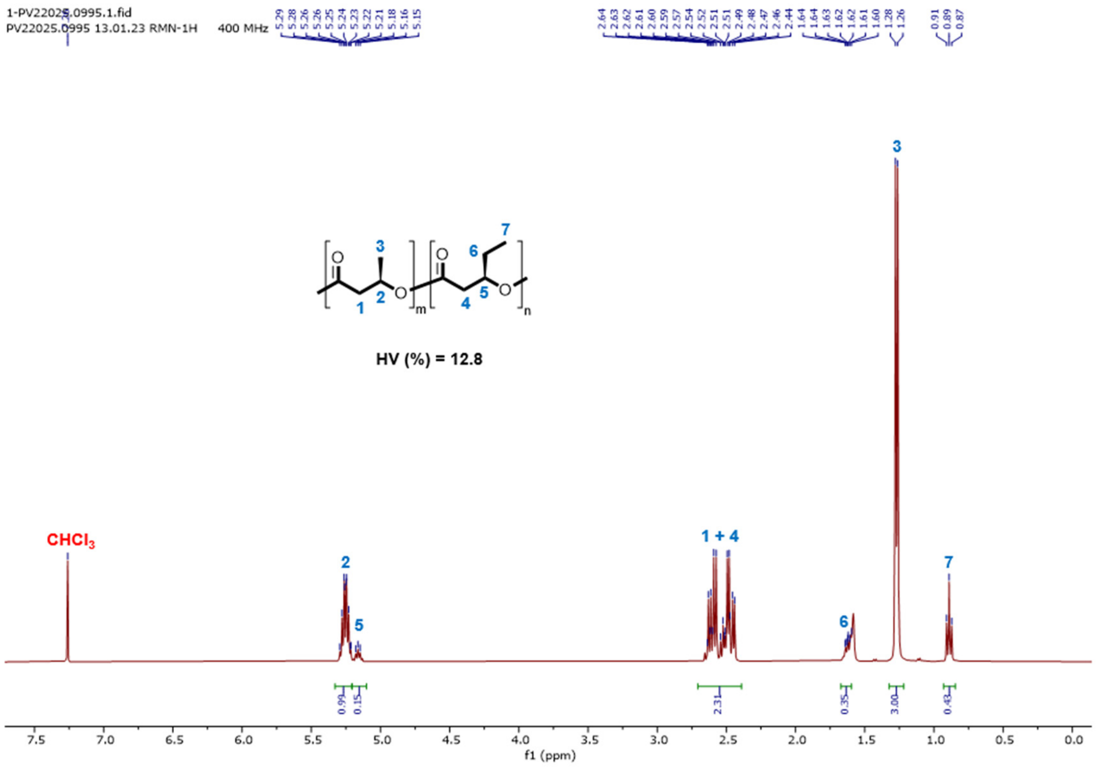

Figure S1. ^{1}H-NMR spectrum (400 MHz, 25 °C) of the sample Control

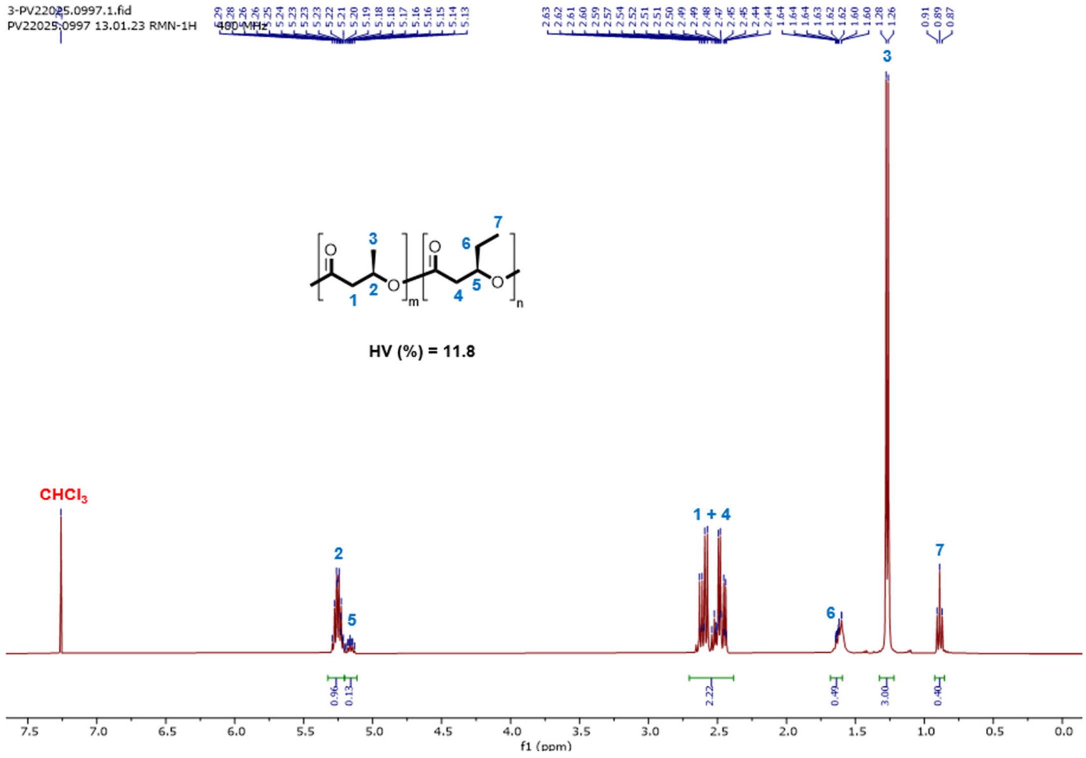

Figure S2. ^{1}H-NMR spectrum (400 MHz, 25 °C) of the sample Residue 1.

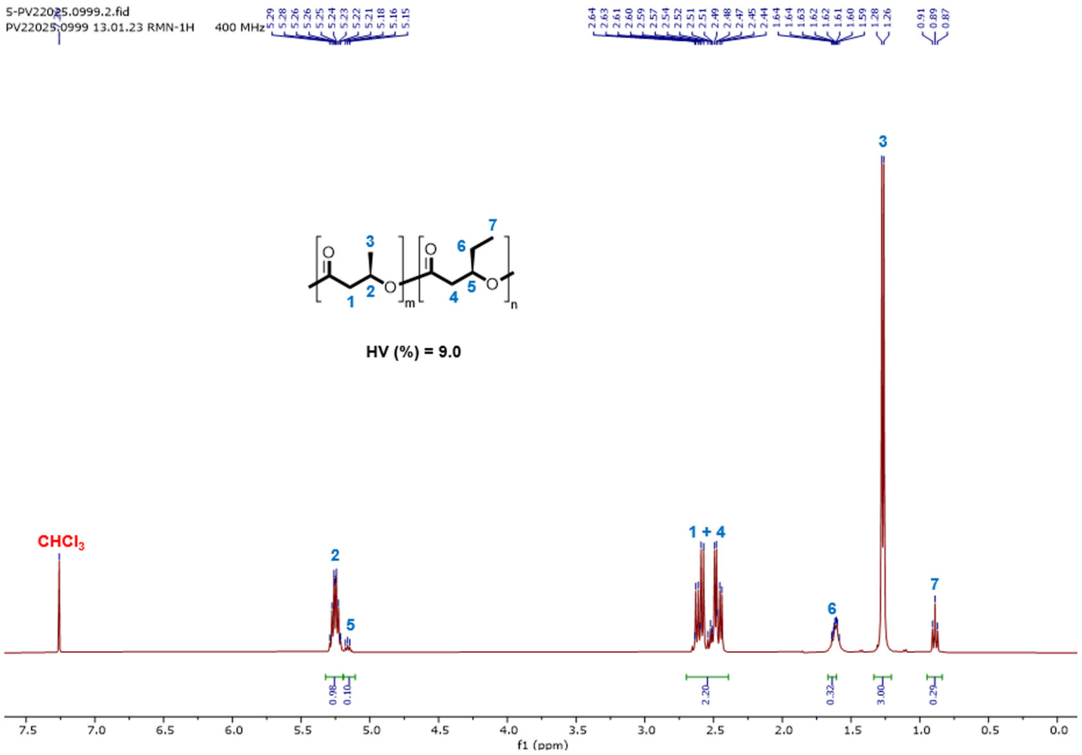

Figure S3. ^{1}H-NMR spectrum (400 MHz, 25 °C) of the sample Residue 2.

DSC curves

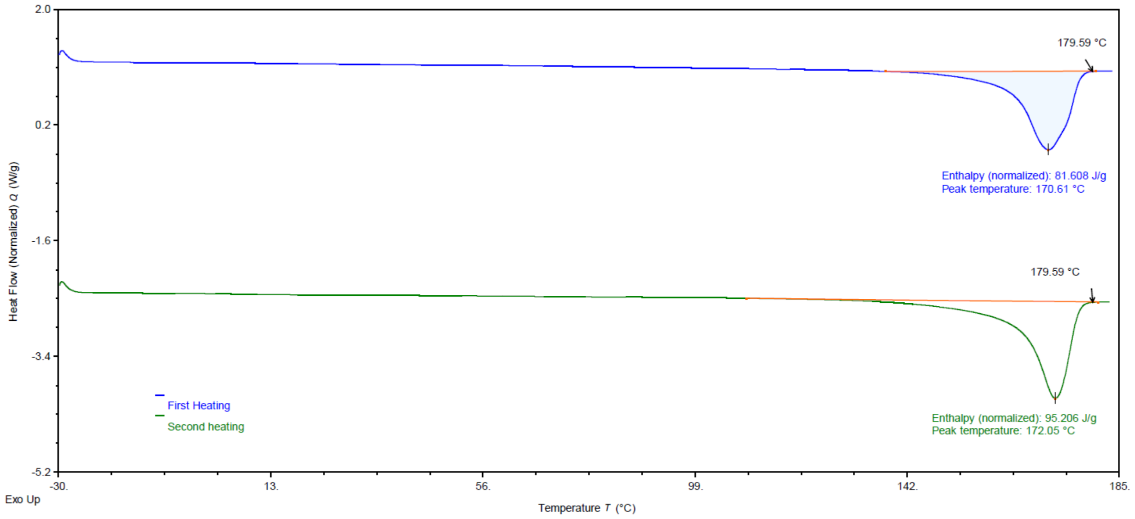

Figure S4. DSC curves of the sample PHBV Y1000P.

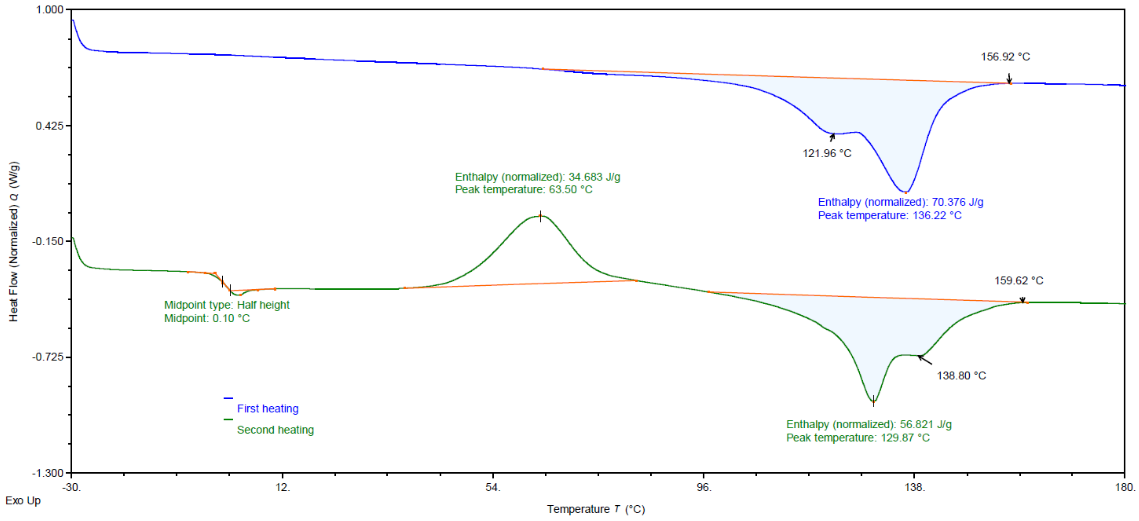

Figure S5. DSC curves of the sample Control

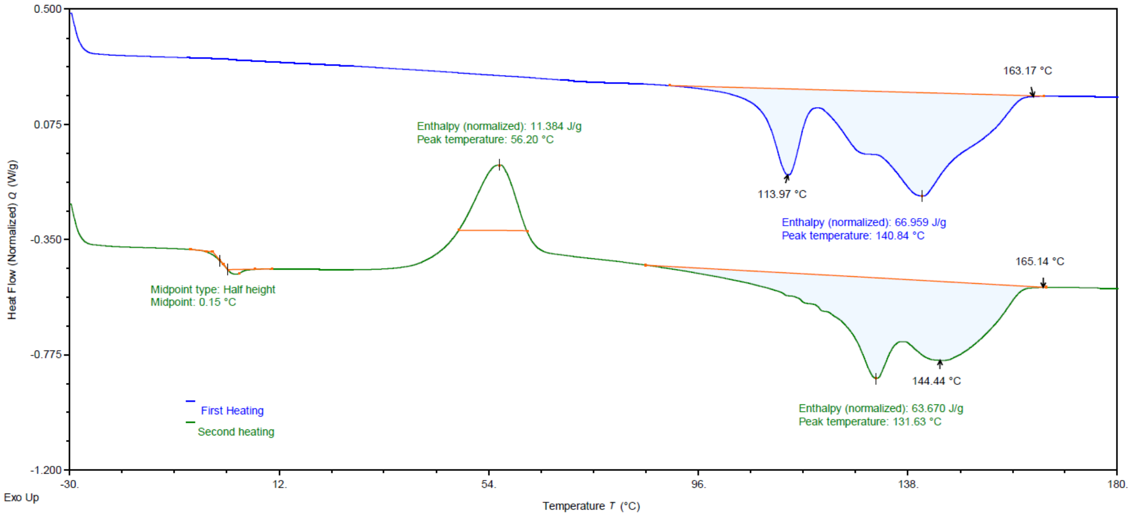

Figure S6. DSC curves of the sample Residue 1.

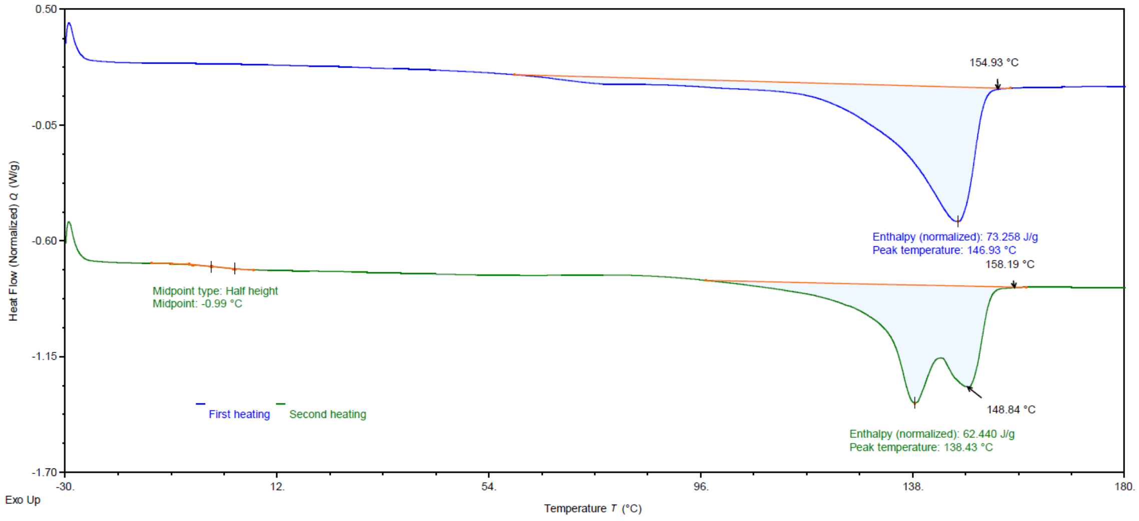

Figure S7. DSC curves of the sample Residue 2.

TGA thermograms

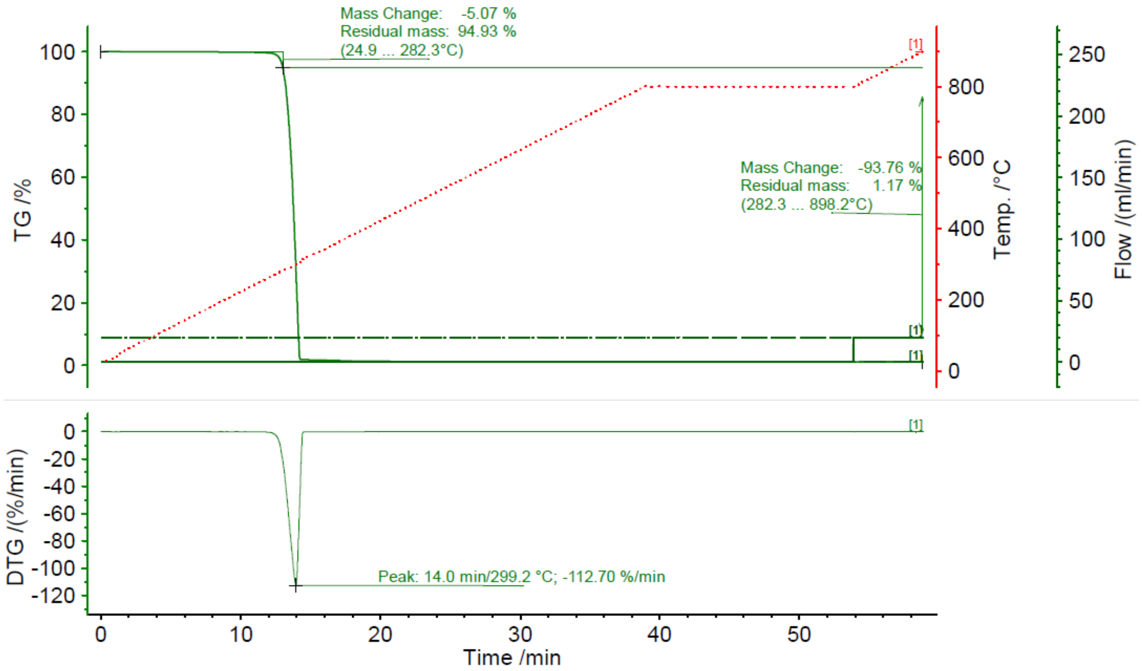

Figure S8. Thermogram of the sample PHBV Y1000P.

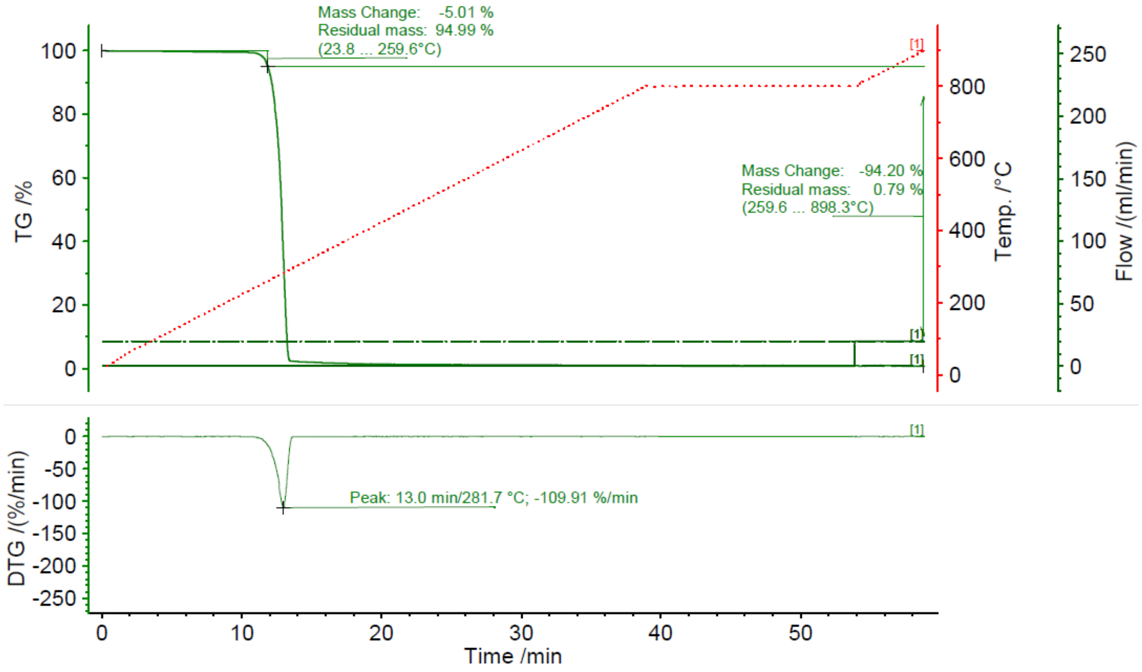

Figure S9. Thermogram of the sample Control.

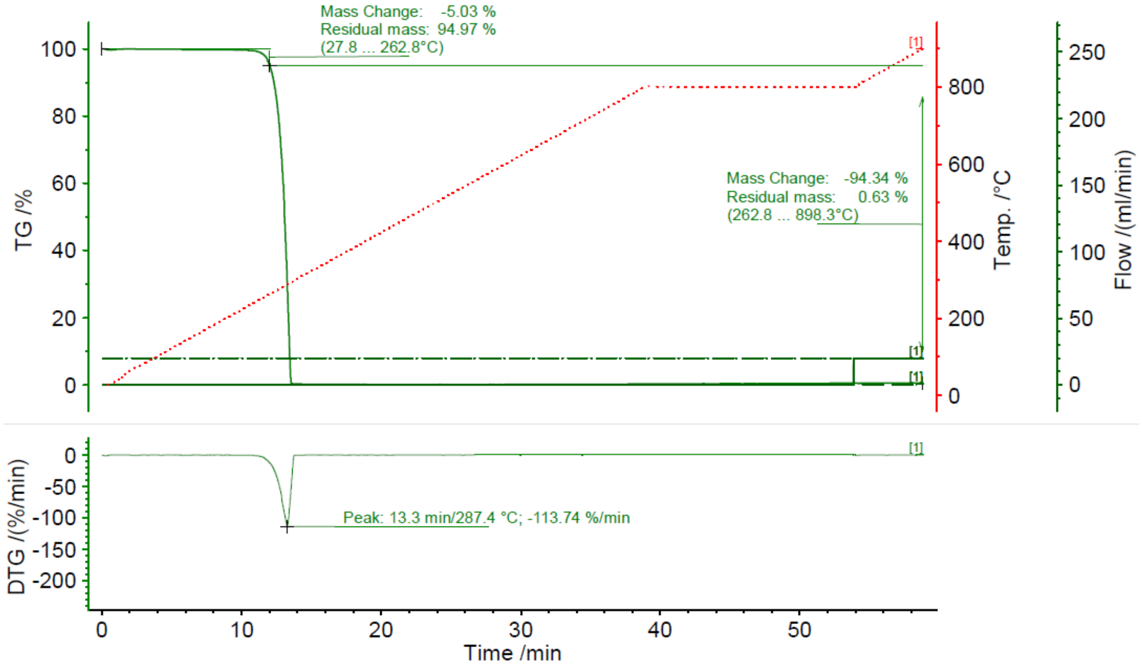

Figure S10. Thermogram of the sample Residue 1.

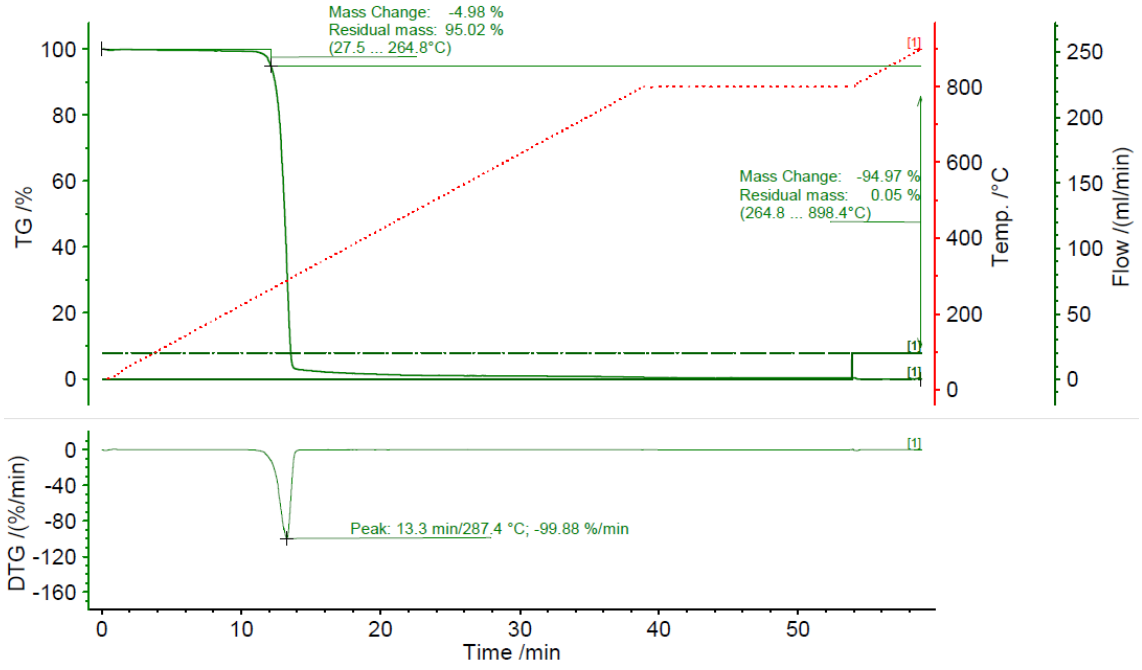

Figure S11. Thermogram of the sample Residue 2.
